# Gender and its association with cardiac defects in down syndrome population at Children Hospital & Institute of Child Health, Lahore, Pakistan

**DOI:** 10.12669/pjms.40.3.7346

**Published:** 2024

**Authors:** Areiba Haider, Sarah Khan, Raafea Tafweez, Muhammad Yaqoob

**Affiliations:** 1 Areiba Haider, MBBS, M-Phil. Assistant Professor of Anatomy, Department of Anatomy, King Edward Medical University, Lahore, Pakistan; 2 Sarah Khan, MBBS, M-Phil. Assistant Professor of Anatomy, Department of Anatomy, King Edward Medical University, Lahore, Pakistan; 3 Raafea Tafweez, MBBS, M-Phil, FCPS, PhD. Professor of Anatomy, Department of Anatomy, King Edward Medical University, Lahore, Pakistan; 4 Muhammad Yaqoob, MBBS, MCPS, PhD. Associate Professor of Genetics, Department of Genetics, The Children’s Hospital & Institute of Child Health, Lahore, Pakistan.

**Keywords:** Down syndrome, Congenital cardiac defects, Gender

## Abstract

**Objective:**

The objective of this study was to determine the frequency of different congenital cardiac defects co-existing in karyotypically proved Down syndrome population. It also highlighted the association between gender and pattern of congenital cardiac defects and gender as a risk factor.

**Methods:**

A cross sectional comparative study was done in the Department of Genetics, Children Hospital Lahore in the year 2017. A total of 160 patients were subjected to karyotypic analysis through blood test for determining the type of Down Syndrome and Echocardiography of all established cases was performed for determining presence and types of congenital cardiac defects. Results were evaluated in terms of establishing co-existence of various cardiac phenotypes in Down Syndrome cases.

**Results:**

In karyotypically proven 160 cases of Down syndrome, 58.1% of Down Syndrome cases and 88.2% of Down Syndrome with Congenital Cardiac Defects presented in infancy. The odds ratio (OR) suggested that females are 1.72 times more likely to experience a cardiac effect compared to males. Female gender was potentially associated (p-value 0.07) with occurrence of Patent Ductus Arteriosus (47.8%), whereas VSD (Ventricular Septal Defects) was most prevalent (41.1%) in males. Patent Ductus Arteriosus + Atrial Septal Defects (44.4%) was the commonest cardiac defect in female cases. The combined data for pattern of cardiac anomalies showed no significant association with gender, as indicated by a p-value of 0.990.

**Conclusion:**

The study concluded that most of Down syndrome cases and Down syndrome with congenital cardiac defects present to the hospital in infancy. Female cases are more prone to develop cardiac defects as compared to males. The manifestation of PDA (Patent Ductus Arteriosus) was significantly associated as an isolated anomaly in females and VSD (Ventricular Septal Defects) as isolated anomaly in males. Patent Ductus Arteriosus tend to co-exist most with ASD (Atrial Septal Defects) in female cases. Gender was not established as a risk factor for affecting the pattern of cardiac defects.

## INTRODUCTION

The survival prospects and life span of people with Down Syndrome has improved over the years, but there are multiple challenges^1^, of them congenital cardiac defects are the most important to consider as they seek treatment more than those with cancer or cystic fibrosis. Almost 13% in childhood and 23% of adult Down Syndrome population succumb to death due to this defect making it the second most potent cause of mortality.^2^,^3^ Male gender is considered to be more prevalent in cases of Down Syndrome. The male predominance is explained by models for joint segregation of chromosome-21 and chromosome Y in spermatogenesis. Female gender faced a 12% higher risk of congenital heart defects compared to males.^4^,^5^

The variety and severity in manifestation of congenital cardiac defects is explained from it being monogenetic to being a multifactorial disorder. The malformations most prevalent in Down Syndrome are the valvulo-septal defects.^6^ Increased gene dosage in Down Syndrome i.e., having an additional chromosome at Chromosome-21 leads to triggering of extra transcription pathways and additional protein, resulting in faulty septo-morphogenesis of heart.^7^,^8^

The reduced expression of TWIST1, a transcription factor that helps in valve development and epithelial-mesenchymal transition (EMT) to the overexpression of genes such as RYR2, NCX, which renders cardiomyocyte-specific phenotype in T21 cardiomyocytes.^9^

The critical regions found on chromosome-21 are more of modulators rather than causative factors.^10^ The present study will determine various cardiac phenotypes manifesting concurrently in Down Syndrome patients and the role of gender of Down syndrome cases in effecting their manifestation in terms of patterns and prevalence. This will provide basis for genetic analysis of such cases in various racial groups and hence determining the pathophysiologic genetic basis of this for management of disease severity.

## METHODS

In this cross sectional comparative study a total of 160 cases of Down Syndrome were assessed. The technique of non-probability purposive sampling was followed. An informed consent was taken from the guardians of the Down Syndrome individuals registered in Genetic Department of Children Hospital, Cardiac Surgery Department of Children Hospital, who had nine out of 14 selected phenotypic features characteristic of Down Syndrome. The gender and age at the time of presentation was recorded.

### Ethical Approval

It was obtained from the Institutional Review Board of King Edward Medical University, Lahore no. 679/RC/KEMU, dated August 24, 2022.

The selected individuals were willing to participate in the study and were below 15 years of age. Sample size was calculated using Single population proportion formula (Z2*(p)* (1-p)/ (e) 2) formula, where z = 1.28(for 80% confidence level), P= prevalence of congenital cardiac defects in Down Syndrome as 45%, e (margin of error) = 0.05, giving a sample size of 160 cases. Data collected, entered and analyzed by the SPSS version 20.0. Data for Age was described using Mean and Standard Deviation. Data for presence of congenital heart defects, subtypes of congenital heart defects and subtypes of Down syndrome was reported by using frequencies and percentages. Chi-square test and binary logistic regression is used to determine gender as a risk factor.

The relevant personal information and physical examination was done in a specified room with comfortable and warm environment and in the presence of either guardian or parent. All the physical parameters were recorded on a predesigned performa, which was developed in the light of features given in Smiths recognizable pattern of malformations.^11^ Karyotyping of all cases was done in order to confirm Down Syndrome. Those whose karyotypic analysis came normal were excluded from the study.

The Cytogenetic Lab of the Genetic Department of Children Hospital was utilized. For karyotyping, a blood sample of 2ml was drawn and immediately transferred to sterile vacuette heparin tube. After half an hour, the blood sample was transferred to a labeled culture flask and later on moved in an incubator for 72 hours at 37°C for harvesting. By the end of 72 hours, 100 µl (0.1ml) Colcemid was added to arrest the cells in metaphase of cell division. Repeated cycles of adding fixative, centrifugation and discarding supernatant were performed several times till fluid became milky due to cell pellet formation. Slides were stained by Giemsa stain for g-banding. Chromosomes were arranged in pairs by their size, length of chromosome and placement of centromere with the help of a software called MAC TYPE-4. The autosomes pairs were numbered from largest (number-1) to smallest (number-22). Any deficient or extra copy of sex chromosome was specially looked for. Multiple metaphase spreads of cases were viewed and analyzed. After the confirmation of aneuploidy, echocardiography of all cases of Down Syndrome was done for the detection of cardiac defect by a specialist pediatric cardiologist.

## RESULTS

The 39.4% of 160 cases of Down Syndrome were diagnosed for a cardiac defect and 58.1% of the total Down Syndrome individuals who presented to the Genetic department for cytogenetic analysis were infants. Although the hospital is a tertiary care center for children but the number of neonates presented to the center were only 11.25%. Eight neonates, six young children and only one child with Down syndrome presented with congenital cardiac defects. The remaining all were infants of which 76.2% were below twelve months of age, (Table-I).

**Table-I:** Distribution of down syndrome cases with cardiac defect according to age of presentation.

Age (months)	Cardiac Defect					

	Yes		No		Total	

	N	%	n	%	N	%
≤ 12.0	48	76.2	49	50.5	97	60.6
12.1 - 24.0	8	12.7	20	20.6	28	17.5
24.1 - 36.0	5	7.9	12	12.4	17	10.6
36.1 - 48.0	0	0.0	1	1.0	1	0.6
48.1 - 60.0	1	1.6	2	2.1	3	1.9
>60.0	1	1.6	13	13.4	14	8.8

Total	63	100.0	97	100.0	160	100.0

Chi-sq = 14.94, P-value= 0.01.

Of the 160 cases there were 94 males (58.8%) and 66 females (41.2%). Thus, the male to female ratio was found out to be 1.42:1. Of all the female cases with Down syndrome 47% had congenital cardiac defects, whereas 34% of male cases had congenital cardiac defects.

The results indicated that, among males, 58.8% did not manifest a cardiac defect, while 33.2% did. For females, 53.0% did not have a cardiac effect, while 47.0% did. Binary logistic regression showed that females have 1.72 times higher odds of experiencing a cardiac defect as compared to males, although this result is not statistically significant (p-value = 0.101). Similarly, the chi-squared test suggested a weak association between gender and cardiac effect, but this association is not statistically significant (χ 2value = 2.71, p-value = 0.099), (Table-II).

**Table-II:** Association between gender and cardiac defect.

	Congenital Cardiac Defect			χ 2 value	P-value	Binary logistic Regression	

Gender	No	Yes	Total	2.71	.099	OR (95%CI)	p-value
Male	62(63.9%)	32(50.8%)	94(58.8%)			Reference	
Female	35(36.1%)	31(49.2%)	66(41.3%)			1.72(.901-3.27)	.101

Total	97(100%)	63(100%)	160(100%)				

The results showed that four males (16.7%) and 11 females (47.8%) had PDA (Patent Ductus Arteriosus). The p-value is 0.071, suggesting a potential but not strong significant association. Followed by VSD (Ventricular Septal Defects) 41.7%, ASD (Atrial Septal Defects) 20.8% in male cases. The p-value was insignificant for the rest of the defects i.e., isolated and combined, indicating no significant role of gender in determining or affecting the pattern of occurrence of the cardiac defects in Down syndrome population, (Table-III).

**Table-III:** Pattern of congenital cardiac defects and its association with gender.

	Gender			p-value

Isolated Anomalies	Male n (%)	Female n (%)	Total n (%)	
PDA	4(16.7)	11(47.8)	15(31.9)	.071
VSD	10(41.7)	7(30.4)	17(36.2)	.467
ASD	5(20.8)	2(8.7)	7(14.9)	.739
CAVSD	4(16.7)	2(8.7)	6(12.8)	.414
TOF	1(4.2)	1(4.3)	2(4.3)	1.0
Total	24(100)	23(100)	47(100)	.884
***Combined Anomalies***				
PDA +ASD	2(28.6)	4(44.4)	6(37.5)	.414
PDA +VSD	2(28.6)	2(22.2)	4(25)	1.00
PDA +TOF	0(0)	1(11.1)	1(6.3)	n.a
PDA +CAVSD	0(0)	1(11.1)	1(6.3)	n.a
VSD+ASD	2(28.6)	1(11.1)	3(18.8)	.564
CAVSD+ASD	1(14.3)	0(0)	1(6.3)	n.a
Total	7(100)	9(100)	16(100)	.617
Overall	31(49.21)	32(50.79)	63(100)	.990

## DISCUSSION

In our study the age of presentation was quite variable. Infants were present in the highest number 58.1%, followed by young children 23.12%, whereas neonates made only 11.25 %. The mean age of presentation for the cases of Down Syndrome was 24.3 months. However, Azman et al. went through the case records of 149 Down Syndrome patients in Malaysia with mean age of presentation 10.6 months.^12^ However, it is imperative to note the infants made the largest group (88.2%), followed by 10.6% in young child category. From this finding we concluded that Down Syndrome individuals with congenital cardiac defect were presented and diagnosed in infancy mostly.

In the present study the male: female ratio was reported to be 1.42:1. This finding is consistent with most of the international studies. Chandra et al., studied 1102 cases of Down Syndrome in the University of Madras and found this ratio to be 1.41:1.^13^ Kovalena a Russian researcher, took up 55 epidemiological studies and came up with the conclusion that the explanation for male gender predominance might be due to joint segregation of chromosome-21 and Y chromosome in spermatogenesis as both belong to the G group of chromosomes. This can also be explained as chromosome-21 non-disjunction during 2^nd^ meiotic division caused by Y chromosome bearing spermatozoa during fertilization.^14^

Our study shows, that the female cases had a predisposition to suffer from a cardiac defect. Our results are consistent with the findings of Sofie Bergstrom et al. who reported in 2016 by a population-based cohort study on 2588 infants with Down Syndrome in Sweden, that the females are 12% more prone to have a congenital cardiac defect as compared to males.^15^ In contrast, Somasundaram and Ramkumar reported that males suffered from cardiac defects more than females.^16^ Further studies should be taken up to determine the pathophysiology behind this. It will definitely help to understand the multifactorial etiology of the cardiac defects in Down Syndrome.

Female Down syndrome cases had highest percentage of PDA (Patent Ductus Arteriosus) whereas VSD (Ventricular Septal Defects) were more prevalent in males along with ASD (Atrial Septal Defects) and CAVSD (Complete Atrioventricular Septal Defects). Which is in contrast to the findings of Ghmaird et al.^17^ Freeman et al. reported that ASD (Atrial Septal Defects) was more prevalent in females.^18^ Santoro et al. reported that Atrioventricular Septal Defects, Atrial Septal Defects, and Ventricular Septal Defects were significantly more frequent in females Down syndrome patients.^19^ Morris et al. reported a higher frequency of CAVSD(Atrioventricular Septal Defects) in females.^20^ An important observation that has come to light in the present study is that Atrial Septal Defects has the highest predisposition to occur as double cardiac defects, the most frequent association is PDA( Patent Ductus Arteriosus )+ASD(Atrial Septal Defects) occurring in 44.4% of female cases of Down Syndrome. Several studies contradict this result. 21

Also female gender may have some association with occurrence of PDA (Patent Ductus Arteriosus) but does not significantly influence the occurrence of pattern of cardiac defects as p-value came out to be 0.99. Diogenes et al. in a meta-analysis in 2017 reported significant association of CAVSD (Complete Atrioventricular Septal Defects) with female gender^22^,^23^ Mughal et al. in 2020, reported 53.8 % male, while 46.2% female cases with cardiac defects. Although it supports our results of having infants as the highest age group of hospital presentation yet the phenotypic associations of cardiac defects largely contradicted our results.^24^ Rehman Y et al. in 2022 at pediatric department of Peshawar Institute of Cardiology, supported the same finding as Mughal.^25^ Aziz et al. only inducted 18 Down syndrome cases which limit the generalization of results.^26^

When gender was considered as a risk factor for causation of cardiac defect, the odds ratio was 1.72, suggesting that females may be more likely to experience a cardiac defect as compared to males but this association is not significant. Therefore, while there may be a slight indication that gender plays a role as a risk factor in causing cardiac defects, more data or further analysis may be needed to confirm any significant association. Uludağ Alkaya et al. has established a significant association between female gender and cardiac defect. 27 Taura M.G. et al. reported that gender was not associated with causation of cardiac defects in Down syndrome.^28^

The present study has highlighted the spectrum of cardiac anomalies appearing in Down Syndrome pool of Pakistan, both in isolated and complex phenotypes. The results will help in assessing the prognosis, improving the genetic counselling and public information facilities and probing on the molecular level the multifactorial nature of trisomy 21and the mechanisms through which they work.

### Limitations

The basis of mechanisms of risk factors like gender, needed to be explored at a molecular level. Also, various pathophysiological basis of manifestation of specific cardiac phenotype in Down Syndrome population of Pakistan which were beyond the scope of the present study.

## CONCLUSION

Most of Down syndrome cases and Down syndrome with congenital cardiac defects present to the hospital in infancy. Female cases are more prone to develop cardiac defects as compared to males. The manifestation of PDA (Patent Ductus Arteriosus) was significantly associated as an isolated anomaly in females and VSD (Ventricular Septal Defects) as isolated anomaly in males. Patent Ductus Arteriosus tend to co-exist most with ASD (Atrial Septal Defects) in female cases. Gender was not established as a risk factor for affecting the pattern of cardiac defects.

### Author`s Contribution:

**AH:** Designed, data collection, statistical analysis, preparation of manuscript, editing of manuscript and responsible of study.

**SK:** Conceived and designed the research, final approval of manuscript.

**RT:** Did statistical analysis and preparation of manuscript.

**MY:** Did karyotypic analysis and review.
